# Observations on the Effects of Opium Applied Externally

**Published:** 1801-12

**Authors:** M. Ward

**Affiliations:** Surgeon to the Manchester Infirmary


### *Obfervations on the Effects of Opium applied externally. By* M. WARD, *Surgeon to the Manchester Infirmary.*

"In the introduction of new modes of treatment, it is incumbent on the medical practitioner, to be fedulouſly cautious, not only that he founds his trials on juft analogies, but that he conducts them with impartiality, and records, with faithfulnefs, their good or ill fuccefs."

*Medical and Phyfical Journal, for July,* 1799, *p.* 445.

IN the latter part of my firft paper on the effects of Opium applied externally, I took the liberty of fuggefting fuch inference ences as appeared to be deducible from the experience I had then had of their effects; namely, 1. That opium, when diligently applied externally so as to be absorbed by the lymphatics, has powerful effects in allaying irritation, removing spasm, and procuring sleep. 2. That it is capable of producing these happy effects, where the exhibition of it internally had not the same salutary operation. 3. That this mode of introducing it into the system may be resorted to with advantage, when it cannot be given internally, or when it will not stay upon the stomach.*

Considering the above as incontrovertible facts, I could not avoid indulging a hope, that some benefit might probably arise from this mode of introducing opium into the system in Hydrophobia and Tetanus; and the more I reflected on the subject, the stronger was my conviction that the misery and danger attendant on these fatal maladies could not be increased by this practice; which is more, I fear, than can be said of the present mode of treatment, in Hydrophobia especially. It is true, I was unable to adduce positive evidence in support of my opinion; but the following considerations seemed sufficient to shew there was nothing improbable in the idea I entertained, and that it was not so wholly destitute of foundation in rational theory as might at first sight appear. With this view, I mentioned, 1. The strong indications in favor of employing opium in these diseases; 2. The frequent inability of swallowing either food or medicine; 3. The violent (and sometimes fatal) spasms which are excited by almost every effort of that kind; † 4. ‡ The peculiar advantage should opium prove an antidote to the canine virus of introducing it by the same system of vessels which convey the virus into the circulation; 5. The inefficacy of every plan of treatment hitherto tried.

After endeavouring to obviate such objections as might be supposed to arise, from its appearing probable that the modus operandi is the same, whether opium be administered internally, or applied externally, I ventured to propose a plan of treatment which seemed less objectionable than those in common use, and more conformable to some of the leading circumstances of the diseases.

That the task I undertook to perform was very imperfectly executed is sufficiently evident; it is therefore not surprising, that my endeavours to demonstrate the absurdity, and even

* Medical and Physical Journal for July 1799, p. 447.

† A powerful motive for discontinuing the attempts to cure by internal means.

‡ This important observation was suggested to me by Dr. Percival.

cruelty, of perfifting in that hackneyed routine of practice in hydrophobia, which experience has repeatedly fhewn to be only calculated to harrafs and diftrefs the patient, and to difappoint and difgrace the medical practitioner, have not been attended with all the fuccefs I could have wifhed.

I conceive however, that much has been done in the interval which has elapfed, towards preparing the way for trying the effects of opiate frictions in hydrophobia, by the unequivocal and fatisfactory demonftrations which have been given of the anodyne and antifpafmodic properties of opium when applied externally, in the courfe of the lymphatics, by means of long continued friction. Various cafes have been recorded where it was thus introduced, and was found to exert its falutary virtues, fo as to produce the moft beneficial confequences, (and without occafioning thofe inconveniences which often arife from its internal ufe, efpecially in large dofes,) after the fame medicine, both alone, and joined with other antifpafmodics, tonics, &c. had been exhibited internally with little or no benefit, or had been rejected altogether;* and though the modus operandi of opium applied externally has not been completely afcertained, we fhall hardly be inclined to reject the important facts we are now acquainted with, merely on that account.

Hitherto my attempts to afcertain the primary operation of opium applied externally, on the pulfe, have not been fuccefsful. Patients afflicted with fpafmodic or convulfive difeafes being ufually timid and apprehenfive, are not favourable fubjects for the purpofe; but I have reafon to believe opium acts more directly and fimply as a fedative, when applied externally, than when given internally; and I think it is principally, if not entirely, owing to this difference in the modus operandi, that the fuperior advantages of applying it externally, in certain cafes are to be attributed.

Several paffages occur in the places juft referred to, which, though not decifive, ftrongly corroborate this idea. In the cafe of Mr. G. P. (Medical Journal for July 1799, p. 441) calmnefs was the firft effect of the opiate frictions, which was fucceeded by fleep, a return of appetite; and the firft time his pulfe was examined, it was found reduced from 100 to 80; and in that of Mrs. ———, (p. 443,) "I found her in a ftate of high delirium without fever. Having remarked, that under fimilar circumftances, the internal exhibition of laudanum aggravated

* Medical and Phyfical Journal for July, 1799, p. 441—9. Ditto for Auguft, 1799, p. 6—8. Ditto for September, 1799, p. 103 and 107. Ditto for February, 1800, p. 102. Ditto for July, 1801, p. 17. Ditto for November, 1801, p. 387, 432, and 433.

vated the affection of her head, probably by producing inebriation, I regarded this as a peculiarly favourable opportunity of trying the ufe of opium externally. I therefore directed, &c. In a few hours fhe was obferved to become confiderably calmer. The like inunction was repeated at night." The day following, "I found her perfectly compofed and rational. She had enjoyed much refrefhing fleep. Her pulfe was regular, her heat natural, and a gentle perfpiration had taken place foon after the firft inunction."

Another cafe is related, (p. 444) where its fedative operation appears ftill more evidently; and in that of John Jackfon, (Medical Journal for Auguft 1799, p. 7,) its fedative power is equally apparent. "June 12. He found means in the night to extricate himfelf from the ftrait waiftcoat, and has been fo noify the two laft nights, as to difturb the patients in the neighbouring wards: He never ceafes talking; his eyes are blood-fhot; a conftant tremulous motion prevails in every part of his frame; tongue furred; pulfe 120. June 13. He flept well the whole of the laft night; the tremor is gone, and he is eafy, compofed, and rational; pulfe reduced from 120 to 88. The ftrait waiftcoat was taken off. He has no complaint, except a flight mazinefs in his head. Two portions of liniment, each containing half a drachm of opium, were applied."

Mr. Henry fays, (Medical Journal for Sept. 1799, p. 104, "She now, with a degree of obftinacy which I feldom witneffed, protefted againft taking any medicine, or even any appropriate food. She was finking faft; her pulfe very rapid and feeble, and fhe had a tendency to delirium. Wine, which in this ftate feemed highly requifite, fhe pofitively rejected; and I defpaired of being able to fucceed by internal means; I therefore refolved to try the external application of opium. The following ointment was directed, &c. This application feemed to have fome fedative effect, and was therefore repeated in the evening. The good effects were each time more evident, fleep almoft conftantly followed the application of the ointment, though no more laudanum was given internally; her pulfe, and the intenfity of heat, were gradually reduced, the diarrhœa was ftopped, &c."

In the fame Number, (p. 107.) a ftriking proof is recorded of the good effects of opium applied externally in gangrene. It was not prefcribed till after the failure of "opium internally both in the folid and liquid form, with as much bark and wine as the patient's ftomach would bear, which fhe took a week without much abatement of the pain; the ulcer ftill fpreading; her ftomach rejecting every thing fhe took, &c. I directed, &c. The next morning fhe was eafier, and the ulcer did not appear appear to have ſpread. The frictions were continued five days longer, with an ounce of the tincture of opium each day; during which the pain left her, and the ulcer looked better every day, &c." (Mr. Barlow had before ſaid, the ulcer had a gangrenous appearance; the pain was exceſſive, and, as ſhe expreſſed herſelf, ſhot from her thumb to her heart.) It is worthy of remark, that the ulceration was ſpreading when the firſt portion of opium was applied, and that its progreſs was immediately arreſted: And this effect of opium is alſo clearly exemplified, in a very intereſting caſe, publiſhed in December 1799, by Mr. Docker, Surgeon to the Forces at Deal. As it eſtabliſhes this point, I ſhall beg leave to tranſcribe it.

"John Baylis, of the third regiment of Guards, was received into the military hoſpital at this place, from Holland, as a venereal patient.

"Upon examination, I found a conſiderable ſphacelation in the groin, from a bubo which had been opened ſome time. It was then rapidly increaſing; and from the general ſtate of his health, I had every reaſon to fear a fatal termination. The part was ordered to be fomented, and the carrot poultice applied; he had cordials, with bark and opium preſcribed, and as much wine as he could take; but, notwithſtanding, the ſphacelation ſtill continued to advance. Oct. 12. This morning I found him extremely ill; the attendants thought he would have died in the night. His ſtomach rejected every thing he took; his pulſe could ſcarcely be counted; a diarrhœa had come on, and his groin preſented a moſt ſhocking appearance. I concluded a few hours would have terminated his exiſtence. It now occurred to me, that opium, introduced by friction, would probably be attended with advantage, and I determined to try it as the laſt reſource. I accordingly directed the following to be immediately rubbed into the thighs and legs. Tinct. opii ʒij. Camph. ℥ij. Ol. oliv. ℥ij. Vitel ovi q. s. M.

"I viſited him a ſhort time afterwards, and found him, I thought, more compoſed and leſs languid, and he had taken ſome wine without ſickneſs. I directed the ſame quantity to be uſed again, and to be repeated in the courſe of the night, with the addition of one drachm of Tinct. Opii. October 13. This morning evidently better; had paſſed the night with ſome ſleep, and the inflammation about the edges of the ulcer conſiderably abated; the diarrhœa had alſo ſtopped. I directed three drachms of Tinct. Opii to be uſed three times in the courſe of the day. I ſaw him in the evening; he had taken a good deal of wine, and ſome ſoup, without any ſickneſs, and his pulſe was better; four drachms of Tinct. Opii were ordered to be uſed during the night. 14th. This morning he appeared in a progreſſive greffive ftate of recovery, though he had flept but little, and perfpired profufely; but he had a regular ftool, and the inflammation about the edges of the ulcer had ftopped, and its furface looked lefs gangrenous. I directed four drachms to be rubbed in every five hours. 15th. A good deal of compofed fleep during the night; the inflammation and fpreading entirely ftopped; the flough feparated from the found parts, and the ulcer now looked quite clean. He has taken a little wine during the laft twenty-four hours, and eat fome fifh with appetite; the friction continued. 16th. Slept the greateft part of the night, and awoke greatly refrefhed; friction continued. 17th. The carrot poultice omitted, and the ulcer treated with fimple dreffings; the friction continued, and he was directed to take fome infufion of bark. 19th. As the bark had created naufea, I ordered it to be omitted; and as his health and appetite now rapidly improve, I diminifhed the opium one third. 24th. He has now a very good appetite, and the ulcer heals rapidly; the opium diminifhed one half. 27th. The opiate friction entirely left off, and he is directed to take the decoction of bark, with elixir of vitriol. Nov. 13th. The ulcer is now almoft healed, and he is, in other refpects, in perfect health." Medical Journal for February 1800, p. 102—4.

Here, the fedative power of opium in diminifhing the irritability, fenfibility and mobility of the fyftem, and the difeafed actions confequent thereon, is exceedingly evident. On the 12th, the fphacelation ftill continued to advance, and the pulfe could fcarcely be counted. On the morning of the 13th, the inflammation about the edges of the ulcer was confiderably abated, the diarrhœa had alfo ftopped; and in the evening, his pulfe was better: (from which I conclude, that whatever other alteration was obfervable in the pulfe, its frequency was diminifhed). On the 14th, the inflammation about the edges of the ulcer had ftopped, and its furface looked lefs gangrenous. 15th. The inflammation and fpreading were entirely ftopped; the flough was feparated from the found parts, and the ulcer looked quite clean. — A remedy capable of frequently producing thefe effects, has long been a defideratum in medicine; and I have great hopes, that opium, applied externally, will, in fome degree, fupply this defect; attention being given to the employment of fuch other means as may tend to promote the object in view.

I fhould feel myfelf greatly indebted to Mr. Docker, if he would have the goodnefs to reply to the following queries, through the medium of the Medical and Phyfical Journal. Did each portion of liniment contain half an ounce of camphire and two two ounces of oil? Have any fyphilitic fymptoms appeared fince the bubo was healed? Has any mercury been employed?*

A cafe of great importance occurs in the Medical Journal for July 1801, p. 17. It is on the external ufe of opium in hiccup, and is addreffed to the Editors of the Medical and Phyfical Journal.—After premifing fome particulars, which it will be unneceffary to introduce here, Mr. Gapper tells us, "On the 19th he was feized with a hiccup, the moft violent, diftreffing, and inceffant I ever witneffed; it continued, without even ceafing a fingle minute, for near *eighty hours*, before any relief was obtained. The pulfe during this period was fmall, and fluctuated from 120 to 130 in a minute. I was not remifs in employing whatever remedies I judged moft likely to be of fervice. Mufk, afafœtida, amber, æther, volatiles, and opium were given, varioufly combined, at fhort intervals, and in confiderable dofes. A large blifter was applied to the region of the ftomach, and fomentations to the feet. At the end of the above mentioned period, not the leaft abatement could be perceived, the hiccup continuing as loud, as violent, and as inceffant as ever. I began now to defpair of my patient; when, as every thing elfe had failed, it occurred to me to employ opium by friction, in the manner I had feen it recommended in your ufeful Journal in cafes of delirium attendant on Typhus fevers. I therefore ordered, &c. When I faw him the next morning, the liniment had been twice ufed, and I was much pleafed to find with fome effect. He was much more compofed than on the preceding night; and though the hiccup was ftill as frequent as ever, yet it was lefs violent and loud; the pulfe was alfo reduced to 112. Encouraged by thefe flattering circumftances, I ordered a continuance of the liniment. On the morning of the 22d he was in much the fame ftate as on the preceding day; but in the evening, I was happy to find that intermiffions, though fhort ones, had taken place. On the next day the intermiffions were increafed in frequency, and were extended to the length of half an hour or more, and the pulfe was reduced to 90. The liniment was continued as before. From this time the hiccup became lefs frequent and violent, and the intermiffions longer, until the 25th, when it wholly left him. The remainder of the cafe, as not being connected with the fubject, it is unneceffary to relate; it is fufficient to fay that my patient ultimately recovered." "During the

* I have lately employed camphire along with opium, externally, in a cafe of Typhus Fever, with a view to its fedative, antifeptic, and antifpafmodic qualities. The effects were beneficial; but not more fo than I have repeatedly feen take place from the employment of opium alone.

the uſe of the liniment he took no other medicine than the ſimple ſaline draught; and ſo far from the bowels becoming conſtipated under its uſe, it was not even neceſſary to give him any aperient medicine during its exhibition. It occaſioned a conſiderable degree of drowſineſs, but not in any degree to excite the leaſt alarm. The quantity of Tinct. Opii uſed was two ounces in the ſpace of little more than three days; * a quantity which could not have been exhibited in any other way without the moſt imminent hazard and danger. Such a wonderful effect as is here related, naturally leads the mind to ſpeculate on the modus operandi, and to diſcover why a certain quantity of opium may be conveyed into the ſyſtem through the abſorbents, not only without detriment, but with great and decided advantage, which, if admitted into the ſtomach, would probably occaſion death. I ſhall not enter into ſuch a diſquiſition, as many and repeated experiments, and much reflection, are neceſſary to give a ſatisfactory explanation; I ſhall only be happy if, in ſimilar circumſtances, it may prove as efficacious in other hands as it has done in mine."

This caſe applies with peculiar force in ſupport of the idea I entertain of the mode of action of opium mentioned above. There is no ambiguity in the ſtatement; every neceſſary information is given of the ſtate of the patient, both before and after the uſe of the opiate frictions. †

In the Medical Journal for Nov. 1801, a caſe well worthy of being preſerved, on account of the important facts it contains, is addreſſed to the Editors, by Mr. White of Bath, who concludes as follows. "What is very remarkable, ſhe never was ſick, nor vomited after the firſt friction, but fell aſleep *immediately* on the application of the opium, and ſlept ſome time. The fomentation and friction were continued a few days, gradually decreaſing the quantity of opium; and as her ſtomach could bear it, a little wine was frequently given. It may be obſerved, that not only the vomiting ceaſed, but the diarrhœa alſo; and ſhe had no complaint afterwards, except a ſlight pain of her legs. Her pulſe was reduced to 96 on the third day, which before was exceedingly quick." "She is recovering her ſtrength very faſt."

I conceive it will be impoſſible to explain the facts juſt re-

* More than this quantity was uſed in the ſame ſpace of time in the caſe of John Baylis.

† I once ſaw a ſimilar inſtance of Singultus, where this was the principal diſeaſe. Every thing proved ineffectual, and the patient died. Had I been at that time acquainted with the powers of opium applied externally, I ſhould not have heſitated to have recommended it.

lated, by ſuppoſing the modus operandi of the medicine to be the ſame in the two different ways of introducing it.*

After the many facts which have been adduced, could any doubts be entertained of opium being more efficacious and ſalutary in ſome diſeaſes, when externally applied, than when given internally, the caſe of Betty Richards, related by Mr. Jenkinſon, (p. 432 of the Medical Journal for November, 1801,) could not fail, I think, to remove them.

From a ſingle inſtance, no general concluſions can, with propriety, be drawn; otherwiſe, Mr. Boutflower's caſe of Tetanus (p. 443) would ſeem to eſtabliſh the ſuperior utility of opiate frictions in that diſeaſe; but I would rather wait till farther experience ſhall enable us to ſpeak with more certainty on a ſubject of ſo much moment. It will be obvious that they cannot be expected to ſucceed in every inſtance, (perhaps in no inſtance) without the aid of ſuch other means as the nature of the caſe and the cauſe producing it, may require.†

*Mancheſter, Nov.* 15, 1801.

[ To be continued. ]

---

### *Remarks on Vaccine Inoculation, by* Mr. Ring, *Member of the Royal College of Surgeons in London.*

To the Editors of the Medical and Physical Journal.

Gentlemen,

In your Journal for October is an article, ſtating, that the Cow-pox appeared in the ſixth century, proving extremely fatal;

---

* It is here taken for granted, that the alterations ſtated to have taken place, are to be attributed principally to the opium, applied externally; not only in this, but in all the caſes, which I have taken the liberty of commenting upon.

† I have lately met with an inſtance of Tetanus, in a girl of eighteen, brought on by a blow on the hypogaſtric region. The inflammatory ſymptoms ran ſo high as to require the repeated applications of leeches, bliſters, antiphlogiſtic purgatives, fomentations, the warm bath, &c. Opium was alſo employed, both internally and externally. The accident happened ſeven weeks ago. When I ſaw her laſt, there was a conſiderable fullneſs and tenſion of the abdomen, and it appeared to me, that matter was forming within the cavity; the ſpaſms had nearly ſubſided for a few days, but at that time they came on more frequently. The opium internally was, I think, rather hurtful, and how far the external application was uſeful, I am unable to ſay.

