# Corrigenda et Addenda

**Published:** 1802-02

**Authors:** 


### CORRIGENDA et ADDENDA.

Vol. vi. p. 482, l. 14, from bottom, for Camph. ℥ij. read Camph. ℥fs.

